# Elevated glioma-related cortical glutamate *in vivo* is associated with *ex vivo* interictal discharges in human brain slices

**DOI:** 10.1093/braincomms/fcaf451

**Published:** 2025-11-17

**Authors:** Bodiabaduge Ashan P Jayasekera, Jehill Parikh, Tamara Modebadze, Anderson Brito Da Silva, Renae J Stefanetti, John Crossman, Damian Holliman, Mohammed A Hussain, Alistair Jenkins, Otto Major, Patrick Mitchell, Claire Nicholson, Nicholas Ross, Dorothy Wallace, Louise Ward, Tim Hodgson, Mark R Baker, Gráinne S Gorman, Andrew Blamire, Mark O Cunningham, Christopher J A Cowie, Fiona E N LeBeau

**Affiliations:** Biosciences Institute, Newcastle University, Newcastle upon Tyne, NE2 4HH, UK; Department of Neurosurgery, Newcastle upon Tyne Hospitals NHS Foundation Trust, Royal Victoria Infirmary, Newcastle upon Tyne, NE1 4LP, UK; Newcastle Magnetic Resonance Centre, Newcastle University, Health Innovation Neighbourhood, Newcastle upon Tyne, NE4 5PL, UK; Biosciences Institute, Newcastle University, Newcastle upon Tyne, NE2 4HH, UK; Translational and Clinical Research Institute, Newcastle University, Newcastle upon Tyne, NE2 4HH, UK; Department of Clinical Neurophysiology, Newcastle upon Tyne Hospitals NHS Foundation Trust, Royal Victoria Infirmary, Newcastle upon Tyne, NE1 4LP, UK; Wellcome Centre for Mitochondrial Research, Newcastle University, Newcastle upon Tyne, NE2 4HH, UK; NIHR Newcastle Biomedical Research Centre, Biomedical Research Building, Campus for Ageing and Vitality, Newcastle upon Tyne, NE4 5PL, UK; Department of Neurosurgery, Newcastle upon Tyne Hospitals NHS Foundation Trust, Royal Victoria Infirmary, Newcastle upon Tyne, NE1 4LP, UK; Department of Neurosurgery, Newcastle upon Tyne Hospitals NHS Foundation Trust, Royal Victoria Infirmary, Newcastle upon Tyne, NE1 4LP, UK; Department of Neurosurgery, Newcastle upon Tyne Hospitals NHS Foundation Trust, Royal Victoria Infirmary, Newcastle upon Tyne, NE1 4LP, UK; Department of Neurosurgery, Newcastle upon Tyne Hospitals NHS Foundation Trust, Royal Victoria Infirmary, Newcastle upon Tyne, NE1 4LP, UK; Department of Neurosurgery, Newcastle upon Tyne Hospitals NHS Foundation Trust, Royal Victoria Infirmary, Newcastle upon Tyne, NE1 4LP, UK; Department of Neurosurgery, Newcastle upon Tyne Hospitals NHS Foundation Trust, Royal Victoria Infirmary, Newcastle upon Tyne, NE1 4LP, UK; Department of Neurosurgery, Newcastle upon Tyne Hospitals NHS Foundation Trust, Royal Victoria Infirmary, Newcastle upon Tyne, NE1 4LP, UK; Department of Neurosurgery, Newcastle upon Tyne Hospitals NHS Foundation Trust, Royal Victoria Infirmary, Newcastle upon Tyne, NE1 4LP, UK; Newcastle Magnetic Resonance Centre, Newcastle University, Health Innovation Neighbourhood, Newcastle upon Tyne, NE4 5PL, UK; Newcastle Magnetic Resonance Centre, Newcastle University, Health Innovation Neighbourhood, Newcastle upon Tyne, NE4 5PL, UK; Newcastle Magnetic Resonance Centre, Newcastle University, Health Innovation Neighbourhood, Newcastle upon Tyne, NE4 5PL, UK; Translational and Clinical Research Institute, Newcastle University, Newcastle upon Tyne, NE2 4HH, UK; Department of Clinical Neurophysiology, Newcastle upon Tyne Hospitals NHS Foundation Trust, Royal Victoria Infirmary, Newcastle upon Tyne, NE1 4LP, UK; Wellcome Centre for Mitochondrial Research, Newcastle University, Newcastle upon Tyne, NE2 4HH, UK; NIHR Newcastle Biomedical Research Centre, Biomedical Research Building, Campus for Ageing and Vitality, Newcastle upon Tyne, NE4 5PL, UK; Newcastle Magnetic Resonance Centre, Newcastle University, Health Innovation Neighbourhood, Newcastle upon Tyne, NE4 5PL, UK; Translational and Clinical Research Institute, Newcastle University, Newcastle upon Tyne, NE2 4HH, UK; Discipline of Physiology, School of Medicine, Trinity College Dublin, Dublin, Ireland; Department of Neurosurgery, Newcastle upon Tyne Hospitals NHS Foundation Trust, Royal Victoria Infirmary, Newcastle upon Tyne, NE1 4LP, UK; Translational and Clinical Research Institute, Newcastle University, Newcastle upon Tyne, NE2 4HH, UK; Biosciences Institute, Newcastle University, Newcastle upon Tyne, NE2 4HH, UK

**Keywords:** epilepsy, electrophysiology, glioma

## Abstract

Gliomas are the most common type of malignant brain tumour and are frequently associated with seizures. Seizures associated with gliomas are frequently refractory to conventional antiseizure medications and constitute a significant cause of morbidity. There is a clear need for a biomarker that will guide resection of the ictogenic areas during neurosurgical procedures to improve seizure control, limit morbidity and achieve better tumour clearance. Several studies have investigated glutamate in the peri-tumoural region using magnetic resonance spectroscopy (MRS), but findings remain inconclusive regarding the association among elevated glutamate, tumour type and seizure history. Furthermore, it is not known if high cortical glutamate would lead to spontaneous interictal discharges (IIDs) *ex vivo*.

In this prospective cross-sectional observational study of patients undergoing surgery for supratentorial gliomas, pre-operative 1D MRS imaging using an echo time (TE) averaged Point Resolved Spectroscopy (PRESS) sequence was performed to quantify glutamate levels in the peri-tumoural region. *Ex vivo* human cortical extracellular local field potential (LFP) recordings from the cortical tissue were used to record the presence or absence of spontaneous IIDs. Metabolite data were compared between cortical regions with spontaneous IIDs (*n* = 6 patients), versus regions with no evidence of spontaneous IIDs (*n* = 14 patients). Our findings provide new metabolic evidence that peri-tumoural glutamate accumulation is strongly linked to spontaneous IIDs (*P* = 0.006), reinforcing its role in glioma-associated seizures. The lack of significant differences in choline to creatine and N-acetyl aspartate to creatine ratios between IID and non-IID regions suggests seizure susceptibility is independent of tumour infiltration or neuronal degeneration. These results highlight MRS as a promising non-invasive tool for identifying metabolically active, seizure-prone cortical regions, which could aid in refining surgical planning. However, further validation is required to determine its utility in predicting clinical seizure occurrence and guiding post-operative seizure management.

## Teaser

Jayasekera *et al.* report that peri-tumoural regions harbouring *ex vivo* evidence of epileptogenicity in the form of interictal discharges have excess cortical glutamate as measured by magnetic resonance spectroscopy compared to peri-tumoural regions which are electrically quiescent, reinforcing the role of glutamate in glioma-related seizures.

## Graphical Abstract

**Graphical Abstract:**
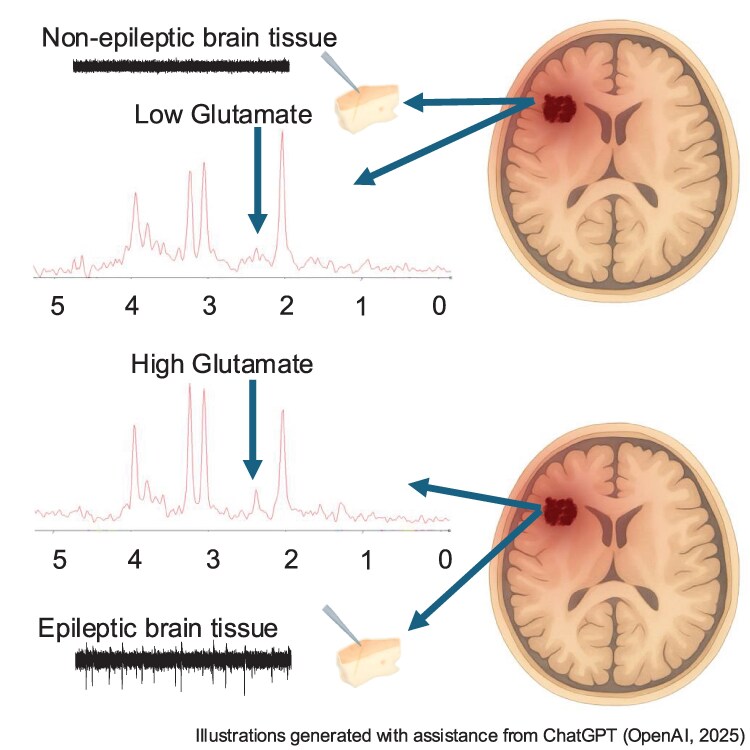

## Introduction

Seizures are a common and debilitating symptom in glioma patients, significantly impacting quality of life and daily functioning.^1^ In low-grade gliomas (LGGs), seizures occur in approximately 60–85% of patients, with 15–50% experiencing drug-refractory epilepsy, particularly in insular or temporal tumours.^2-4^ Glioblastomas, the most aggressive and high-grade gliomas, have a seizure prevalence of 40–60%, with around 40% presenting with seizures at diagnosis and up to 10% developing drug-resistant epilepsy.^3,5^

Surgical resection is the most effective approach for long-term seizure control in glioma patients. The extent of resection is a key predictor of postoperative seizure outcomes, with gross total resection associated with seizure reduction or complete cessation in 80% of cases, whereas subtotal resection results in seizure control in only 53%.^6,7^ However, complete resection is feasible in less than half of LGG cases due to proximity to eloquent cortex and the risk of neurological deficits.^8,9^ Even with gross total resection, 20% of patients continue to experience seizures,^6^ due to epileptogenic tissue extending beyond tumour margins.^8^ There is evidence that resecting cortical tissue beyond the margins of the tumour, i.e. a supratotal resection, leads to better seizure control. Peri-tumoural corticectomy or additional hippocampectomy in temporal low-grade gliomas has demonstrated improved seizure control.^6,8,10,11^ In temporal lobe glioblastoma, an anterior temporal lobectomy leads to better seizure control compared to gross total tumour resection alone.^12^ Whilst the utility of intraoperative electrocorticography (ECoG) mapping remains inconclusive,^13,14^ one study has suggested that seizure control is better in patients undergoing ECoG guided resections of adjacent high risk epilepsy cortical areas compared to patients undergoing gross total resection alone.^15^ Chronic subdural or depth electrode monitoring provides better localization but carries risks, with complications in 25% of cases and 9% requiring additional surgery.^14^

Despite surgical resection, a significant subset of glioma patients continue to experience seizures, highlighting the limitations of current treatment strategies. A recent systematic review reported that while antiseizure medications achieve a ≥50% reduction in seizure frequency in 75–86% of patients, a subset remains drug-resistant.^16^ While newer therapies, such as perampanel, an AMPA receptor antagonist, have shown promise in brain tumour-related epilepsy^17^ pharmacological management alone is often insufficient. Given these challenges, optimising surgical strategies to better target epileptogenic tissue is critical.

A non-invasive method to map epileptogenic cortex preoperatively could enhance surgical planning and improve seizure outcomes. Identifying reliable biomarkers, such as elevated glutamate levels detected via magnetic resonance spectroscopy (MRS), may help delineate seizure-generating regions. While peri-tumoural glutamate elevation has been reported in glioma models,^18-23^ human tissue studies^24^ and MRS research, its relationship to seizure activity remains inconclusive.^25,26^ Some studies report an association between high cortical glutamate levels and seizures,^25^ while others do not.^26,27^ In addition, lower glutamate levels have been found in glioma patients with the isocitrate dehydrogenase (IDH) enzyme mutation compared to IDH wild type glioma patients.^26,27^ When IDH wild type patients were looked at exclusively, the glutamate signal was higher in the seizure group.^26^ Using advanced MRS techniques, such as an echo-time (TE) averaged Point Resolved Spectroscopy (PRESS) sequence, may improve the differentiation between glutamate and glutamine.^28-30^ Other metabolites detectable by MRS, such as N-acetyl-aspartate (NAA) and lactate, may also help predict refractory epilepsy.^25^ Higher choline levels are seen in higher-grade gliomas with increasing cellularity and cell membrane turnover.^31^ Creatine is often used as a reference metabolite with a reduction in NAA thought to reflect dysfunction, or death of neurons.^32,33^

IIDs serve as an established biomarker of epileptogenicity, correlating with seizure onset zones^34-36^ and resecting the IID-generating cortex improves postoperative seizure outcomes.^37^ However, while ECoG can detect IIDs intraoperatively, prolonged invasive monitoring is impractical in glioma surgery. *Ex vivo* cortical slice models have demonstrated epileptiform activity resembling IIDs,^38,39^ offering insights into epileptogenesis.^40-42^ Using a multimodal approach, our objective was to determine whether peri-tumoural MRS glutamate signals correlate with IID-generating cortical regions *ex vivo*. We hypothesized that peri-tumoural regions with elevated glutamate signal on MRS are associated with spontaneous seizure activity in the form of IIDs. We also wanted to establish whether NAA/Cre ratios, a marker of neuronal density,^43^ and Chol/Cre ratios, a marker of tumour burden,^44^ were different between cortical regions that generated IIDs and those that did not. Lastly, we examined whether there were differences in MRS metabolite ratios between patients with and without seizures and IDH mutant status.

A precise, non-invasive method to define tumour resection margins is still needed. While more extensive resection is associated with better postoperative seizure freedom,^45^ it can increase the risk of post-operative disability for patients, which in turn is associated with reduced survival.^46^ Therefore, we need a better understanding of the changes occurring in the peri-tumoural tissue to justify more extensive resection. This study aimed to investigate the relationship between excess cortical glutamate and IIDs *ex vivo* to determine whether MRS, as a non-invasive technique for identification of the seizure-generating peri-tumoural region, had potential for use in surgical planning.

## Materials and methods

### Patient selection and ethics

Patients with gliomas evaluated at the Royal Victoria Infirmary in Newcastle (UK) were recruited into two MRI/MRS studies from 2018 to 2020. Glutamate in Glioma Related Seizures (GGRS) recruited 27 patients with a radiological diagnosis of glioma (grades 2–4) for MRI/MRS and sampling of peri-tumoural cortex for *ex vivo* electrophysiology recordings. Seven patients were excluded as the spectra did not meet the quality criteria. This was approved by the Coventry and Warwickshire NRES committee (REC reference 17/WM/0434). Glutamate dysfunction in Glioma (GDG) recruited patients with low-grade gliomas for MRI/MRS and high-density (128 channel) EEG recordings. Only the MRI/MRS data from this second study were included for this paper. This was approved by Northeast—Newcastle and North Tyneside 2 Research Ethics Committee (REC reference 17/NE/0236). The inclusion and exclusion criteria for both studies were as follows:

#### Inclusion criteria

Patients were only eligible for the GGRS/GDG studies if they met all the following criteria:

1. Males and females over the age of 18 years at the time of screening.
2. Patient must have a proven radiological diagnosis consistent with glioma.
3. Females of child­bearing age must have a negative serum/urine pregnancy test.
4. Capacity to provide informed consent taken before any study-related activities.
5. Ability and willingness to adhere to the protocol, including all appointments.
6. Ability to read and converse in English.

#### Exclusion criteria

Patients were not eligible for the GGRS/GDG studies if they met any of the following criteria:

1. Contraindications to magnetic resonance imaging (implanted cardiovascular and non-cardiovascular devices, electronic implants and metallic debris in the eyes/body).
2. Women with a positive serum/urine pregnancy test.
3. Claustrophobia prevents imaging within an MRI scanner.
4. Patients without the capacity to provide informed consent.
5. Patients unwillingness to adhere to the protocol, including all appointments.
6. Language barriers prevent patients from reading and conversing in English.

Five healthy volunteers were also recruited.

### Magnetic resonance imaging/spectroscopy

MRI and MRS data were acquired on a Phillips Achieva 3 Tesla MRI scanner using an eight-channel head coil. A volumetric T1 study, axial T2 weighted study and axial and coronal FLAIR sequences were acquired in all patients and used to help place the volume of interest (VOI). MRS data were acquired using a 1D chemical shift imaging (CSI) method based on a VOI of 20 mm × 20 mm × 20 mm, which was subdivided by phase encoding into contiguous reconstructed voxels of 20 mm × 20 mm × 5 mm. The excited VOI was placed within the second voxel overlying the cortical region to be biopsied by the surgeon (Supplementary Fig. 1). The region of interest was agreed upon with the operating surgeon prior to surgery as part of the operative corridor to the tumour in question. A TE (echo time) averaged PRESS sequence^30^ was used to measure metabolite signals with TE times of 35, 55, 75, 95, 115, 135 and 155 ms acquired and averaged, which effectively nulls the signal from the glutamine resonances, which overlap with glutamate. Other sequence parameters included the following: TR (Repetition time) 2000 ms; 1024 averages; spectral bandwidth 2000 Hz; scan duration 1 min 36 s per echo time and variable power radiofrequency pulses with optimized relaxation delays (VAPOR) water suppression.^47^ A control VOI was placed in glioma patients in the contralateral hemisphere from the same region of brain. MRI and MRS data were also acquired from healthy volunteers with one VOI in the right hemisphere. An example of the location of the selected VOI in one patient is shown in Supplementary Fig. 1.

MRS data were analysed offline using JMRUI v 3.0. The spectra were hard phased based on the residual water peak and the water peak removed with Hankel Lanczos Singular Values Decomposition Filter. An 8 Hz Gaussian Apodize function was used for visual display and noise reduction. Each TE spectra were visually inspected to ensure there was no residual water peak, no lipid contamination and acceptable baseline variation before summing to obtain the TE averaged spectrum. Metabolite signals were quantified using the Advanced Method for Accurate, Robust, and Efficient Spectral fitting (AMARES), a non-linear least squares quantitation algorithm^48^ using a database of starting values for NAA, glutamate, choline and creatine. A good fit by AMARES was also checked and an acceptable shim with an NAA linewidth of less than or equal to 10 Hz. If the spectra in the voxel of interest did not meet the quality criteria, the next voxel was examined to see if the spectra met the criterion for inclusion in the final analysis. Data were expressed as ratios relative to creatine.

### Neuronavigation guided sampling

Screenshots of the MRS voxel were transferred to the Newcastle Upon Tyne NHS Foundation Trust PACS system and used to plan the placement of a target on Brainlab-compatible T1-weighted images acquired within the trust, using the Brainlab planning station (Brainlab AG, Munich). Intra-operatively, the operating surgeon used the Brainlab Cranial Neuronavigation System to identify the cortical surface overlying the MRS voxel and excise a sample of 1 cm^3^ in volume using sharp dissection only, minimizing handling and limiting where possible the use of bipolar coagulation to maintain tissue viability.

### Human *ex vivo* electrophysiology recordings

#### Collection, slicing and slice maintenance.

After being excised by the operating surgeon, tissue was immediately immersed in cold (4°C) modified human artificial cerebrospinal fluid (aCSF) in mM (180 sucrose, 2.5 KCL, 10 MgSO_4_, 25 NaHCO_3_, 1.25 NaH_2_PO_4_, 10 glucose, 0.5 CaCL_2_, 20 ethyl pyruvate, 2 N-acetyl cysteine, 1 taurine, 1 ascorbic acid, 0.1 Trolox/6-hydroxy-2,5,7,8-tetramethylchroman-2-carboxylic acid, 0.1 aminoguanidine, 0.045 indomethacin, Sigma–Aldrich, Gillingham, UK) and carbogenated (95%O_2_/5%CO_2,_ BUSE gases, West Bromwich, UK). Collected samples were then quickly transferred to the laboratory (<5 min). Any pia or blood vessels on the cortical surface that might hinder slicing were removed by sharp dissection in ice cold aCSF. Tissue was mounted and cut to 450 μm slices using a vibratome (Microm HM 650 V, Thermo Fisher Scientific, Altrincham, UK). Slices were then incubated at room temperature for 30 min in a holding chamber and covered with parafilm in holding aCSF (containing: 126 mM NaCl; 3 mM KCl; 24 mM NaHCO_3_; 1.25 mM NaH_2_PO_3_; 1.2 mM CaCl_2_; 2 mM MgSO_4_; and 10 mM glucose). Slices were then transferred to a standard interface recording chamber at 34–36°C perfused with aCSF containing MgSO_4_ at a lower concentration of 1 mM, at 290–310 mOsm, saturated with 95% O_2_ and 5% CO_2_. Normal aCSF was circulated through the interface chamber with a Gilson minipuls3 pump. The interface chamber was heated to 34–36°C with a water pump heater (FH-16D, Grant Instruments Limited, Beckenham, UK). An infrared thermometer was used to check temperatures throughout the experiments. Slices were left in the interface chamber for 30 min prior to recording. The average time from collection to the first recording was approximately 3 h. All recordings were completed within 12 h.

#### Extracellular recordings

IIDs were defined as a spontaneous paroxysmal field potential deflection with a sharp-wave/spike morphology. Extracellular field recordings of IIDs were conducted in superficial cortical layers using aCSF-filled borosilicate glass electrodes (1.2 mm O.D.×0.94 mm I.D.—Harvard Apparatus Ltd, UK) prepared by pulling into micropipettes (2 MΩ) using a PP-83 puller (Narishige, Tokyo, Japan). Glass electrodes were connected to an extracellular amplifier (EXT-10-2F; Npi electronic GmbH, Tamm, Germany) and mounted in a micromanipulator. Two micromanipulators were on each rig, allowing recordings from two separate slices. Extracellular recordings were amplified with a gain of 500, high-pass filtered at 1 Hz and low-pass filtered at 500 Hz with a NeuroLog system (Digitimer, Letchworth Garden City, UK). Mains noise (50 Hz) was eliminated with a Hum Bug noise eliminator (Digitimer, Letchworth Garden City, UK). Analogue extracellular recordings were then digitized at 5 kHz using an ITC-18 multichannel data acquisition interface (Digitimer, Letchworth Garden City, UK) and recorded using Axograph X software (Axon Instruments, Foster City, USA). Slices were visualized using a NIKON SMZ645 stereomicroscope (NIKON, Tokyo, Japan). In some instances, multi-electrode array recordings were conducted using Buzsaki-style probes (64 electrode; NeuroNexus Technologies, Ann Arbor, Michigan, USA) connected to an Intan RHD2000-series amplifier (Intan Technologies, Los Angeles, California, USA) system. Signals were amplified and digitized (20 kHz) using the Intan system and downsampled for offline analysis of the extracellular local field potential using MATLAB (MathWorks, Natick, Maine, USA).

### Statistical analysis

Statistical analyses were performed on GraphPad Prism Version 10 for Windows, 64-bit (GraphPad Software, Boston, USA). Data were tested for normality using the Shapiro–Wilk test given the small sample sizes.^49^ Parametric data were analysed using the unpaired *t*-test and quoted as means ± standard deviation and displayed as histograms. Non-parametric data were analysed using the Mann–Whitney test and Kruskal–Wallis test and quoted as median with interquartile range (IQR) and displayed as box plots with all the data points, median, minimum and maximum values. A *P* value of less than 0.05 was considered statistically significant and denoted by a single *. *P* values less than 0.01 and 0.001 were denoted by ** and ***, respectively. Illustrations for the graphical abstract were created with assistance from OpenAI’s ChatGPT (GPT-5, image generation tool, 2025).

## Results

### Patient demographics

**Figure 1:**
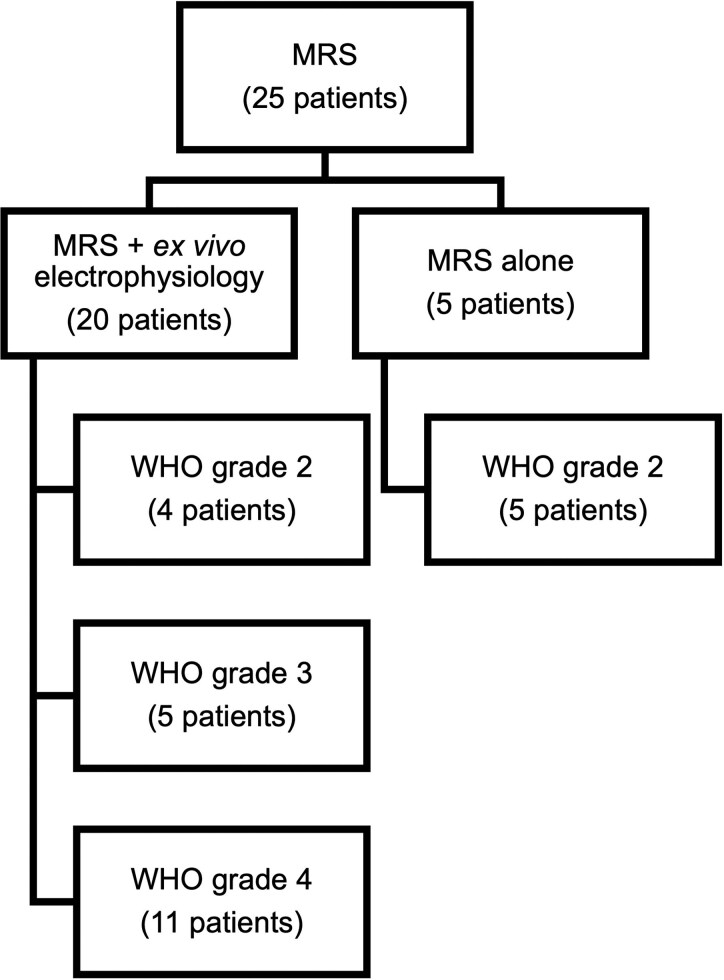
Flow diagram of patient recruitment.

MRS data sets were available from 25 patients with gliomas (Fig. 1). Twenty patients were recruited for combined magnetic resonance imaging (MRI) and MRS acquisition and *ex vivo* electrophysiological recordings. MRI/MRS data sets were available for a further five patients with low-grade gliomas (World Health Organization (WHO) grade 2). Healthy control MRS data were acquired from five volunteers. Summary demographics are listed in Table 1, and individual demographics in Supplementary Table 1. All patients gave full and informed consent.

**Table 1:** Summary demographics by study

	Study		
Patient demographics	MRS + electrophysiology (*n* = 20)	MRS alone (*n* = 5)	Healthy volunteer (*n* = 5)
Average age (years)	52.5 ± 14.5	49.6 ± 17.0	27.8 ± 4.3
Male: female (numbers)	16:4	3:2	3:2
Glioma grade (*n*): grade 2: grade 3: grade 4	4 :5 :11	5: 0:0	n/a
Frontal (*n*)	10	2	1
Insular (*n*)	0	1	0
Parietal (*n*)	2	0	1
Temporal (*n*)	5	1	2
Occipital (n)	3	1	1
On anticonvulsants (*n*): yes: no	13: 7	4: 1	n/a

Demographics of age, gender, glioma grade, location by lobe and history of seizures for each study group, MRS + electrophysiology, MRS alone and healthy volunteers. (n/a = not applicable).

The sample of MRS and *ex vivo* electrophysiology recordings included 4 patients with a WHO grade 2 glioma, 5 with a WHO grade 3 and 11 with WHO grade 4 (Fig. 1, Table 1). The MRS alone sample included five patients with a WHO grade 2 glioma.

The average patient age was older in the MRS + *ex vivo* electrophysiology cohort (average 52.5) than the MRS alone cohort (average 49.6), which is unsurprising given the higher incidence of high-grade glioma in older patients.^50^ There were also more males in both groups, consistent with the higher incidence of glioma in males.^51^ The frequency of pre-operative seizures was similar in both groups (Table 1). More insular tumours were seen in the MRS alone cohort, consistent with the greater involvement of this region in low-grade gliomas.^52^ The MRS + *ex vivo* electrophysiology cohort had more frontal lobe tumours, reflecting the higher grade of gliomas.^53^

### Regions with *ex vivo* spontaneous interictal discharges have higher Glut/Cre ratios than IID-negative regions

**Figure 2:**
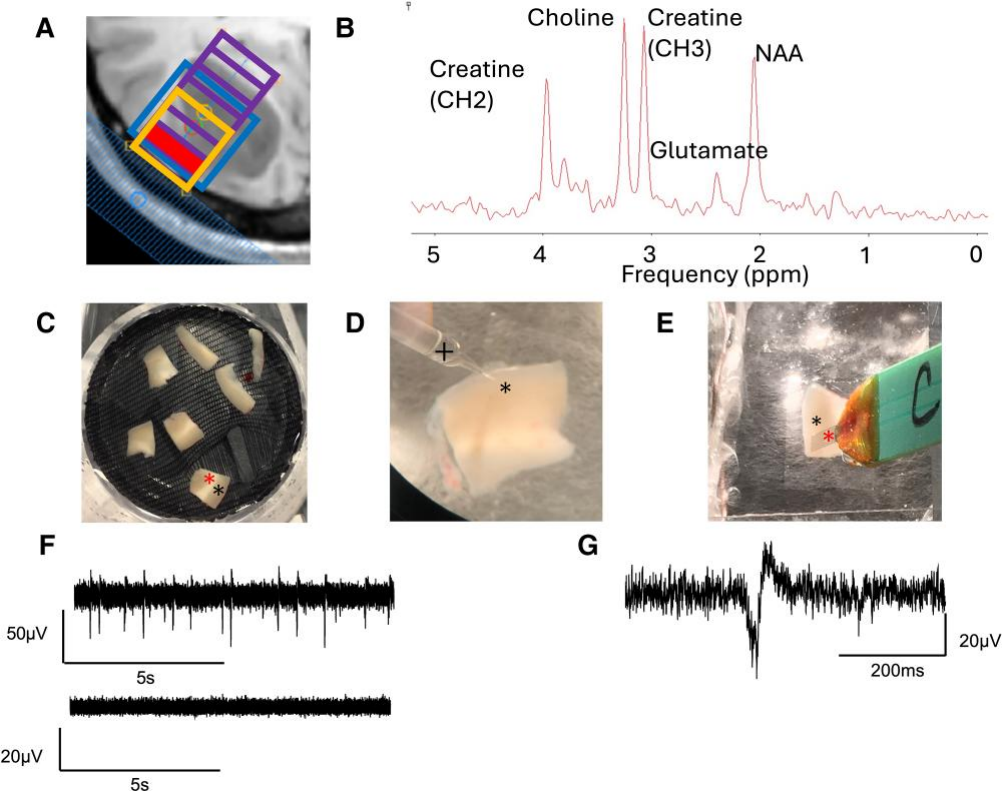
**Methods for peri-tumoural MRS and ex vivo electrophysiology.** (**A**) VOI (orange) within 1-D chemical shift imaging (CSI) volume of interest (purple grid) highlighting in red the cortical area to be sampled within the ‘surgical corridor’. (**B**) MR spectra from VOI with creatine (CH2), choline, creatine (CH3), glutamate and NAA peaks annotated. (**C**) Example of human tissue slices in a holding chamber with preserved cortex (*) and subcortical white matter (*). (**D**) Glass electrode (+) for recording LFPs from superficial layers of cortex (*). (**E**) Silicone MEA (Multi Electrode Array) recording (+) of superficial cortical layers (*). (**F**) An example of extracellular field recordings showing a slice with spontaneous IIDs (top panel) and another slice recording with no spontaneous IIDs, so-called IID-negative slices (bottom panel). (**G**) Expanded trace of single spontaneous IIDs (*) from **F**).

Prior to surgical resection, patients underwent MRS to evaluate Glut/Cre, NAA/Cre and Chol/Cre ratios. Cortical tissue to be used for *ex vivo* LFP recordings was identified as part of the surgical corridor to access the tumour with the operating neurosurgeon. Pre-operatively, a volume of interest (VOI) was selected in that surgical corridor from which to acquire the MR spectrum (Fig. 2A and B). After transportation to the lab and slicing, human cortical slices were then placed in an interface chamber and LFP recordings were made in normal aCSF (Fig. 2C–E) to determine whether spontaneous IIDs were present (Fig. 2F and G).

*Ex vivo* spontaneous IIDs were present in cortical slices from six out of 20 scanned patients; no spontaneous activity was seen in the remaining 14 patients. A case was considered to exhibit epileptiform activity if at least one slice showed evidence of spontaneous IIDs, while cases in which no slices showed any abnormal activity were defined as IID-negative. The IID-positive cohort consisted of cases from the frontal (*n* = 1), temporal (*n* = 2), parietal (*n* = 2) and occipital (*n* = 1) regions. In contrast, samples with no IIDs were from frontal (*n* = 9), temporal (*n* = 3) and occipital (*n* = 2) regions. None of the *ex vivo* slices from any case exhibited spontaneous ictal discharges (IDs).

**Figure 3:**
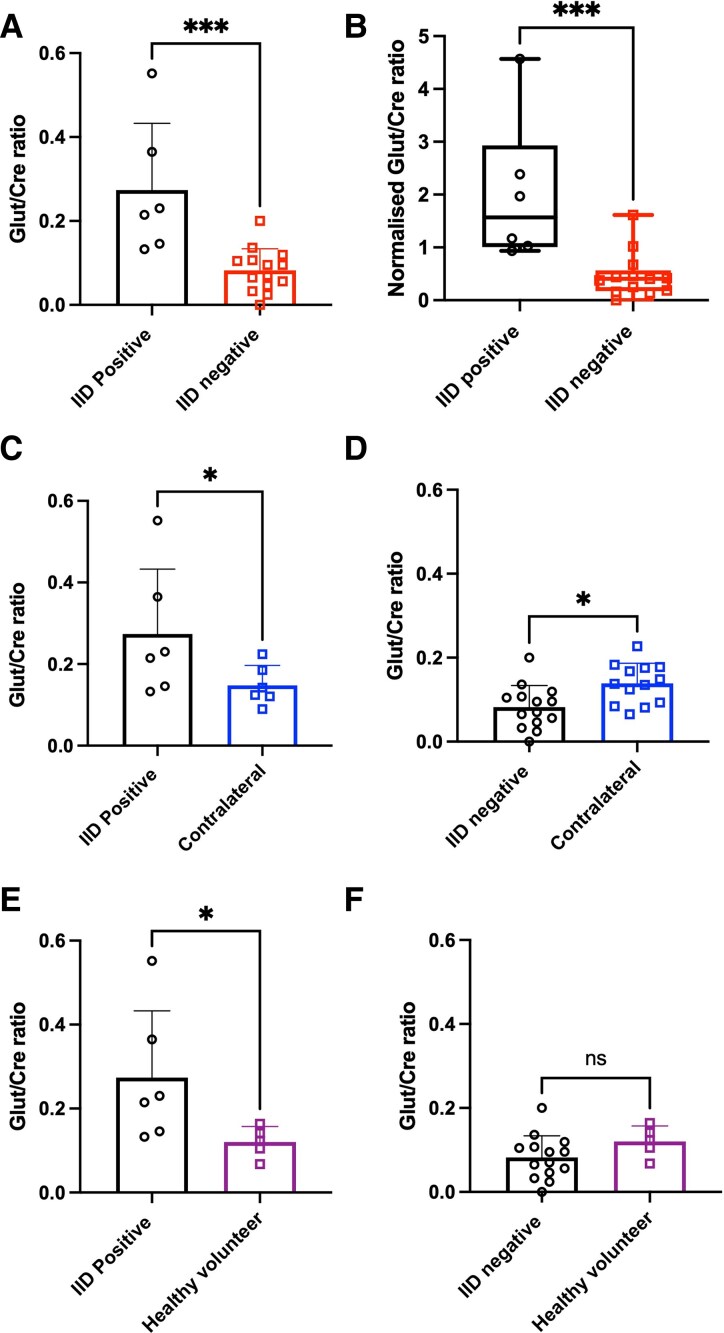
**Comparison of Glut/Cre ratio in IID-positive versus IID-negative regions.** (**A**) Glut/Cre ratios were higher in cortical tissue from cases with spontaneous IIDs (*n* = 6) compared to regions that were IID-negative (*n* = 14, *P* = 0.0006, two-tailed *t*-test). (**B**) Normalised Glut/Cre ratios were higher in regions exhibiting spontaneous IIDs (*n* = 6) compared to regions that were IID-negative (*n* = 13, *P* = 0.0009, Mann–Whitney test). (**C**) Regions with spontaneous IIDs had higher Glut/Cre ratios compared to a control region in the contralateral hemisphere (*n* = 6, *P* = 0.0429, one-tailed *t*-test). (**D**) In contrast, IID-negative regions had lower Glut/Cre ratios compared to a contralateral VOI region in the same patient (*n*= 13, *P* = 0.0183, two-tailed *t*-test). (**E**) Spontaneous IID regions had higher Glut/Cre ratios (*n* = 6) compared to healthy volunteer data (*n* = 5, *P* = 0.0334, one-tailed *t*-test). (**F**) IID-negative regions had lower Glut/Cre ratios (*n* = 14) than healthy volunteer data, but this was not statistically significant (*n* = 5, *P* = 0.1512, two-tailed *t*-test), ***P < 0.001 *P < 0.05 ns *P* > 0.05. Individual data points are Glu/Cre metabolite ratios for each subject.

We found that cortical regions from IID-positive patients had significantly higher Glut/Cre ratios measured *in vivo* compared to cortical regions from IID-negative patients (0.28 ± 0.16 versus 0.08 ± 0.05, *P* = 0.0006, two-tailed *t*-test, Fig. 3A, IID-positive: *n* = 6, IID-negative: *n* = 14). To the best of our knowledge, this is the first time that cortical glutamate levels on MRS have been compared between regions that exhibited spontaneous IIDs *ex vivo* and regions with no IIDs.

To confirm that this was not due to globally elevated glutamate, Glut/Cre ratios measured in the peri-tumoural region were normalized to the corresponding region in the contralateral hemisphere in the same patients. The normalized Glut/Cre ratios remained significantly higher in IID-positive patients compared to IID-negative patients (median = 1.57, IQR 1.01–2.93 versus 0.41, IQR 0.17–0.57, *P* = 0.0009, Mann-Whitney test, Fig. 3B, IID-positive: *n* = 6, IID-negative: *n* = 13). One contralateral region in the IID-negative cohort could not be included for analysis. Additionally, cortical regions from IID-positive patients had higher Glut/Cre ratios compared to their own contralateral volume of interest (VOI) (0.28 ± 0.16 versus 0.15 ± 0.05, *P* = 0.0429, one-tailed *t*-test, Fig. 3C, *n* = 6). In contrast, cortical regions from IID-negative patients had lower Glut/Cre ratios compared to their own contralateral VOI (0.08 ± 0.05 versus 0.14 ± 0.05, *P* = 0.0183, two-tailed *t*-test, Fig. 3D, *n* = 13). Comparison with healthy controls further confirmed these findings. The Glut/Cre ratios in cortical regions from IID-positive glioma patients were significantly higher than those measured in healthy volunteers (0.28 ± 0.16 versus 0.12 ± 0.04, *P* = 0.0334, one-tailed *t*-test, Fig. 3E, IID-positive glioma patients: *n* = 6, healthy controls: *n* = 5). In contrast, cortical regions from IID-negative patients showed lower Glut/Cre ratios compared to healthy volunteer data, though this difference was not statistically significant (*P* = 0.1512, two-tailed *t*-test, Fig. 3F, IID-negative glioma patients: *n* = 14, healthy controls: *n* = 5).

These findings indicate an association between elevated cortical glutamate levels and epileptogenicity in glioma patients, supporting the hypothesis that increased peri-tumoural glutamate is linked to spontaneous IID generation.

### NAA/Cre ratios were similar between regions with spontaneous interictal discharges and IID-negative regions

**Figure 4:**
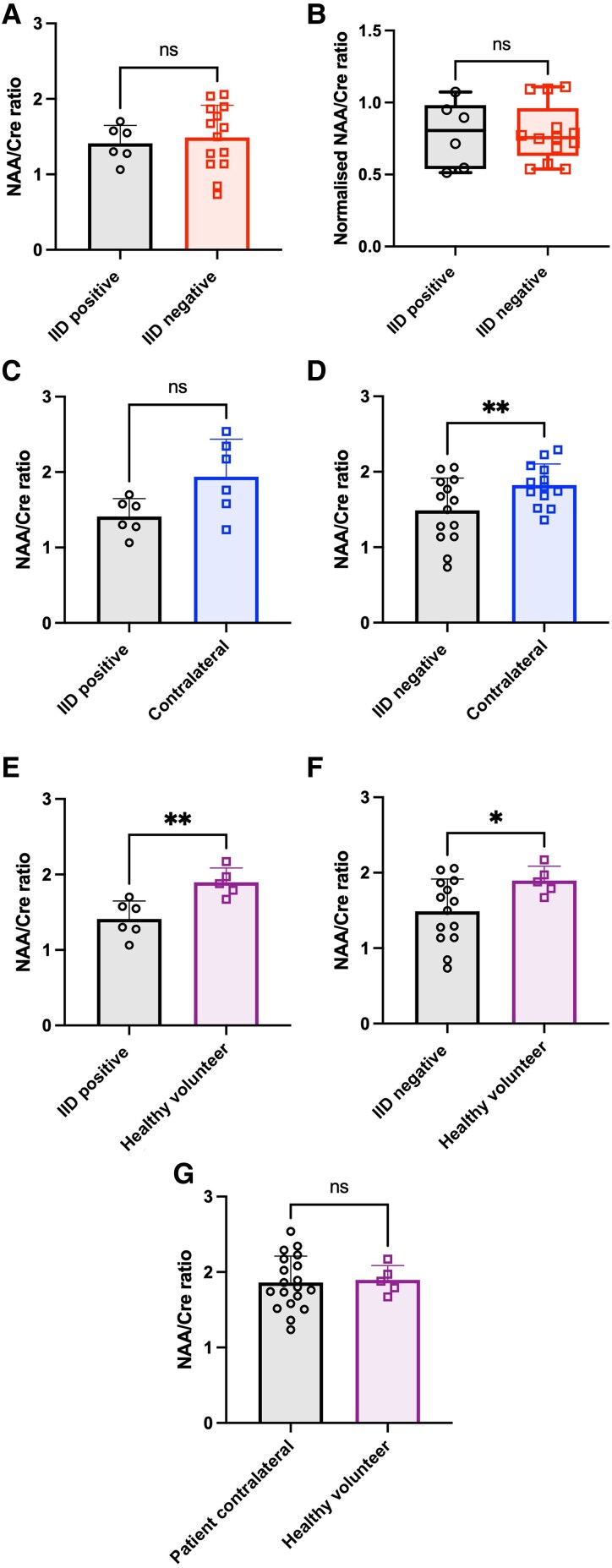
**Comparison of NAA/Cre ratios in spontaneous and IID-negative regions.** (**A**) NAA/Cre ratios were not different between cortical samples with spontaneous IIDs (*n* = 6) compared to cases where slices were IID-negative (*n* = 14, *P* = 0.6828, two-tailed *t*-test). (**B**) Normalized NAA/Cre ratios were not different between regions with spontaneous IIDs (*n* = 6) compared to regions where all slices were IID-negative (*n* = 13, *P* = 0.8148, Mann–Whitney test). (**C**) Regions with spontaneous IIDs had similar NAA/Cre ratios compared to a control region in the contralateral hemisphere (*n* = 6, *P* = 0.0536, two-tailed *t*-test). (**D**) In contrast, IID-negative regions had lower NAA/Cre ratios compared to a contralateral region in the same patient (*n* = 13, *P* = 0.0058, two-tailed *t*-test). (**E**) Regions with spontaneous IIDs had lower NAA/Cre ratios (*n* = 6) compared to healthy volunteer data (*n* = 5, *P* = 0.0050, one-tailed *t*-test). (**F**) IID-negative regions also had lower NAA/Cre (*n* = 14) ratios compared to healthy volunteer data (*n* = 5, *P* = 0.0292, one-tailed *t*-test). (**G**) NAA/Cre ratios were no different between patient contralateral regions (*n* = 19) and healthy volunteer data (*n* = 5, *P* = 0.2383, two-tailed *t*-test) **P < 0.01 *P < 0.05 ns *P* > 0.05. Individual data points are NAA/Cre metabolite ratios for each subject.

The difference in the Glut/Cre ratio outlined above could reflect a greater reduction in the number of neurons in regions that were IID-negative *ex vivo* due to peri-tumoural neuronal destruction.^19^ If this were the case, then we would expect to see a reduction in the NAA/Cre ratio, a neuron-specific metabolite which can act as a marker of neuronal density and health.^43,54^ However, there was no difference in NAA/Cre ratios between regions that generated spontaneous IIDs (mean = 1.41 ± 0.24, *n* = 6) and regions that had no IIDs *ex vivo* (mean = 1.49 ± 0.43, *n* = 14, *P* = 0.6828, two-tailed *t*-test, Fig. 4A). There were still no statistical differences when NAA/Cre ratios were normalized to a VOI acquired from the contralateral hemisphere (Fig. 4B, spontaneous IID regions median = 0.8, IQR 0.54–0.98, *n* = 6, IID-negative regions median = 0.76, IQR 0.63–0.96, *n* = 13, *P* = 0.8148, Mann–Whitney test). Given that these samples have been acquired in peri-tumoural regions where glioma would be expected to infiltrate and destroy surrounding brain, we also compared NAA/Cre ratios within regions that exhibited spontaneous IIDs *ex vivo* (mean = 1.41 ± 0.24, *n* = 6), and contrasted them with a contralateral VOI in the same patient (mean = 1.94 ± 0.50, *n* = 6). However, although the NAA/Cre ratios appeared lower within regions that exhibited spontaneous IIDs *ex vivo* this did not reach statistical significance (*P* = 0.0536, two-tailed *t*-test, Fig. 4C).

In the patients whose *ex vivo* slices were all IID-negative, the NAA/Cre ratios were significantly lower in the sampled region (mean = 1.45 ± 0.41, *n* = 13) compared to the contralateral VOI (mean = 1.82 ± 0.28, *n* = 13, *P* = 0.0058, two-tailed *t*-test, Fig. 4D). Furthermore, NAA/Cre ratios were lower in regions that exhibited spontaneous IIDs *ex vivo* (mean = 1.41 ± 0.24, *n* = 6), compared to healthy volunteer data (mean = 1.90 ± 0.19, *n* = 5, *P* = 0.0050, one-tailed *t*-test, Fig. 4E). Similarly, NAA/Cre ratios were also lower in IID-negative regions (mean = 1.49 ± 0.43, *n* = 14) compared to healthy volunteer data (mean = 1.90 ± 0.19, *n* = 5, *P* = 0.0292, one-tailed *t*-test, Fig. 4F). Interestingly the NAA/Cre ratios were no different between patient contralateral regions (mean = 1.86 ± 0.35, *n* = 19) and data acquired from healthy volunteers (mean = 1.90 ± 0.19, *n* = 5, *P* = 0.2383, two-tailed *t*-test, Fig. 4G), suggesting that the contralateral regions are normal as far as MRS metabolite data are concerned.

Overall, these data suggest that neuronal density is similar between cortical regions that exhibited spontaneous IIDs *ex vivo* and those that were IID-negative, as no significant differences in NAA/Cre ratios were detected. However, neuronal density was reduced in peri-tumoural regions compared to the contralateral hemisphere and healthy volunteers, indicating that neuronal loss is a general feature of peri-tumoural cortex, rather than a distinguishing characteristic of IID-generating regions. This may account for the lower Glut/Cre ratio observed in the IID negative regions compared to the contralateral hemisphere.

### Cho/Cre ratios were similar between regions with spontaneous interictal discharges and IID-negative regions

**Figure 5:**
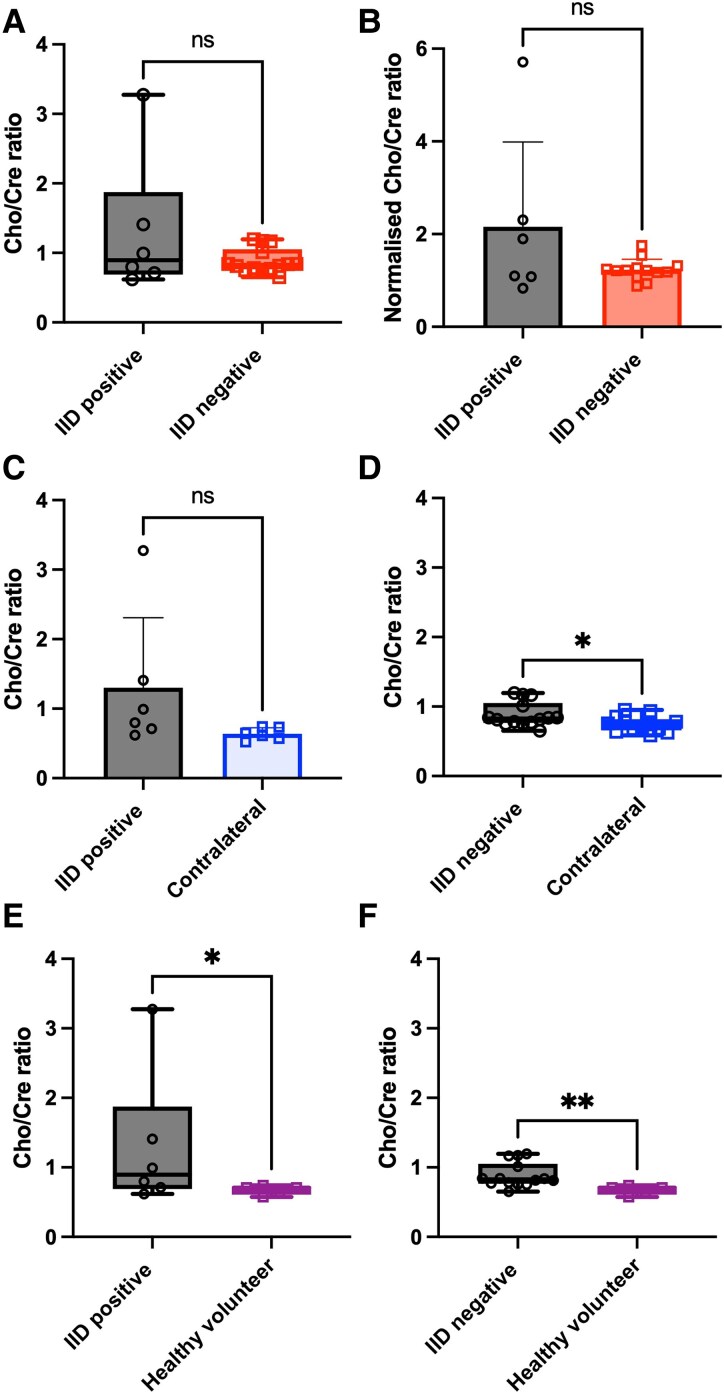
**Comparison of Cho/Cre ratios in IID-positive and IID-negative regions.** (**A**) Cho/Cre ratios were different in regions with spontaneous IIDs *ex vivo* (*n* = 6) compared to regions that were IID-negative (*n* = 14, *P* = 0.9044, Mann–Whitney test). (**B**) Normalized Cho/Cre ratios were not different between regions with spontaneous IIDs (*n* = 6) compared to regions that were IID-negative (*n* = 13, *P* = 0.0816, two-tailed *t*-test). (**C**) Regions with spontaneous IIDs had similar Cho/Cre ratios compared to a control region in the contralateral hemisphere (*n* = 6, *P* = 0.1213, two-tailed *t*-test). (**D**) In contrast, IID-negative regions had lower Cho/Cre ratios compared to a contralateral VOI region in the same patient (*n* = 13, *P* = 0.0398, Mann–Whitney test). (**E**) Cho/Cre ratios were elevated in regions with spontaneous IIDs (*n* = 6) *ex vivo* compared to healthy volunteer data (*n* = 5, *P* = 0.0411, one-tailed Mann–Whitney test). (**F**) Cho/Cre were elevated in IID-negative regions (*n* = 14) compared to healthy volunteer data (*n* = 5, *P* = 0.0012, Mann–Whitney test). **P < 0.01 *P < 0.05 ns *P* > 0.05. Individual data points are Chol/Cre metabolite ratios for each subject.

The Cho/Cre ratio is a marker of lipid cell membrane turnover and an indicator of tumour invasion.^44^ Cho/Cre ratios were no different in regions with spontaneous IIDs (median = 0.89, IQR 0.69–1.89, *n* = 6) versus IID-negative regions (median = 0.83, IQR 0.77–1.05, *n* = 14, *P* = 0.9044, Mann–Whitney test, Fig. 5A), even when normalized to a contralateral VOI (IID-positive mean = 2.15 ± 1.83, *n* = 6; IID-negative mean = 1.23 ± 0.23, *n* = 13, *P* = 0.0816, two-tailed *t*-test, Fig. 5B). Cho/Cre ratios were slightly elevated in the IID-positive regions (mean = 1.30 ± 1.00, *n* = 6) compared to contralateral VOIs (mean = 0.64 ± 0.09, *n* = 6), although this did not reach statistical significance (0.1213, two-tailed *t*-test, Fig. 5C). However, in the IID-negative group, Cho/Cre ratios were significantly elevated (IID-negative median = 0.84, IQR 0.78–1.09, *n* = 13; contralateral median 0.74, IQR 0.66–0.82, *n* = 13, *P* = 0.0398, Mann–Whitney test) (Fig. 5D). The Cho/Cre ratios were also elevated in regions that elicited spontaneous IIDs *ex vivo* (median = 0.89, IQR 0.69–1.89, *n* = 6), compared to healthy volunteer data (median = 0.71, IQR 0.61–0.73, *n* = 5, *P* = 0.0411, one-tailed Mann-Whitney test, Fig. 5E). Cho/Cre ratios were unsurprisingly also elevated in IID-negative regions (median = 0.83, IQR 0.77–1.05, *n* = 14) compared to healthy volunteer data (median = 0.71, IQR 0.61–0.73, *n* = 5, *P* = 0.0012, Mann-Whitney test, Fig. 5F).

Tumour infiltration, as indicated by Cho/Cre ratios, was similar between cortical regions exhibiting spontaneous IIDs *ex vivo* and those that were IID-negative. However, peri-tumoural regions showed significantly higher Cho/Cre ratios compared to the contralateral hemisphere and healthy controls, indicating increased choline metabolism as a general feature of glioma-affected cortex rather than a specific marker of epileptogenicity.

### No metabolic differences in patients with a history of pre-operative seizures versus no seizures and IDH mutant status

Several studies examining glutamate levels have reported conflicting data in relation to whether patients had a history of seizures or not pre-operatively.^25,26,55^ Metabolite differences between low-grade and high-grade gliomas are well established.^56,57^ We therefore also wanted to establish in our dataset whether there was any difference in Glut/Cre, NAA/Cre and Chol/Cre ratios across different patient groups. For this analysis, we have also included additional data from patients with MRS but no *ex vivo* electrophysiology.

**Figure 6:**
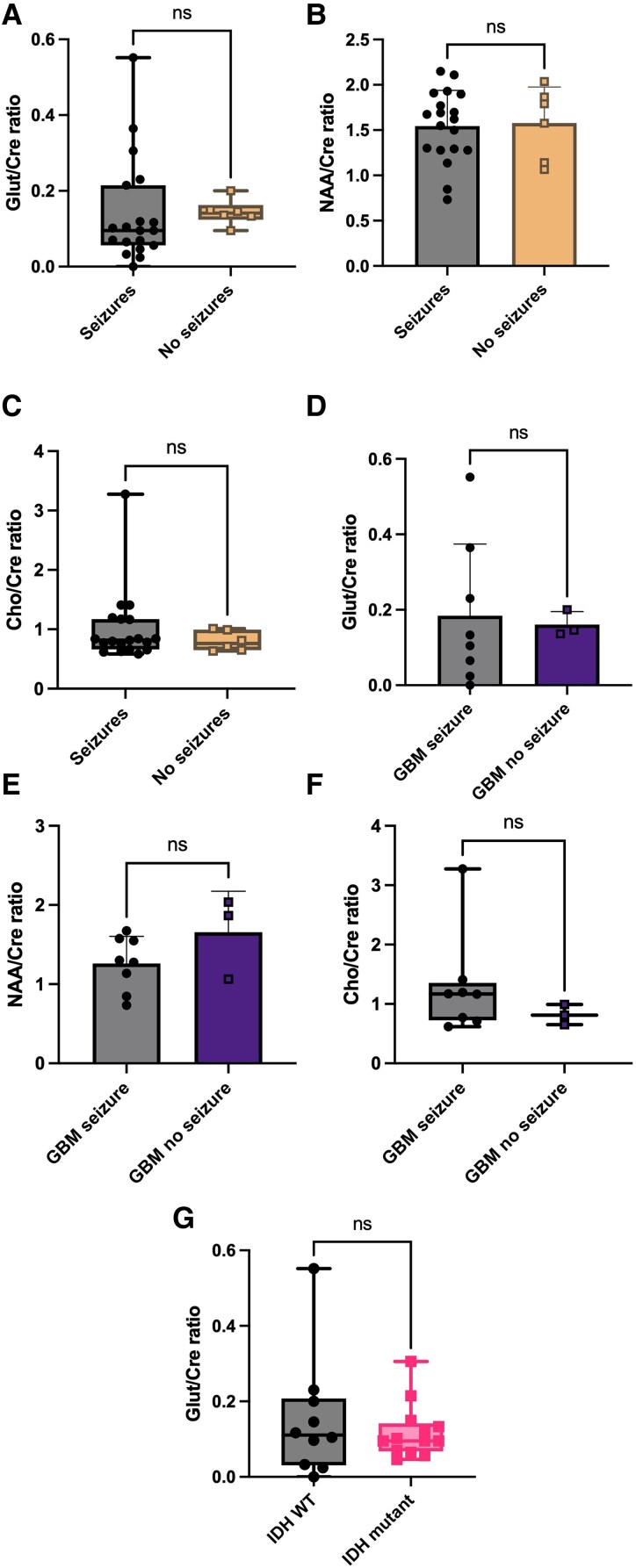
**Metabolic differences between seizure and no-seizure groups.** (**A**) No significant difference in Glut/Cre ratio between the seizure group (*n* = 19) and no-seizure group (*n* = 6, *P* = 0.2161, Mann–Whitney test). (**B**) No significant difference NAA/Cre ratios between the seizure group (*n* = 19) and no-seizure group (*n* = 6, *P* = 0.9976, two-tailed *t*-test). (**C**) No significant difference in Cho/Cre ratios between the seizure group (*n* = 19) and the no-seizure group (*n* = 6, *P* = 0.5430, Mann-Whitney test). (**D**) Glut/Cre ratios were also not different between glioblastoma (GBM) with seizures (*n* = 8) and no seizures (*n* = 3, *P* = 0.8411, two-tailed *t*-test). (**E**) No difference in NAA/Cre ratios between GBM patients with seizure (*n* = 8) and no seizures (*n* = 3, *P* = 0.1686, two-tailed *t*-test). (**F**) No difference in Cho/Cre ratios no different between GBM patients with (*n* = 8) or without seizures (*n* = 3, *P* = 0.3758, Mann–Whitney test). (**G**) No difference in Glut/Cre ratio between IDH WT (*n* = 10) and IDH mutant gliomas (*n* = 13, *P* = 0.8315, Mann–Whitney test). **P < 0.01 *P < 0.05 ns *P* > 0.05. Individual data points are metabolite ratios for each subject.

There were no statistically significant differences in the Glut/Cre ratio (Fig. 6A) between the two groups (seizures median = 0.10, IQR 0.06–0.22 *n* = 19, no seizures median = 0.14, IQR 0.12–0.16 *n* = 6, *P* = 0.1561, Mann–Whitney test). NAA/Cre ratios were also not statistically different between the seizure group (mean = 1.55 ± 0.39, *n* = 19) and non-seizure group (mean = 1.58 ± 0.40, *n* = 6, *P* = 0.8580, two-tailed *t*-test Fig. 6B). Cho/Cre ratios were also not different between the seizure group (median = 0.82, IQR 0.67–1.17, *n* = 19) and non-seizure group (median = 0.76, IQR 0.65–0.10, *n* = 6, *P* = 0.5063, Mann–Whitney test, Fig. 6C).

To assess any impact of tumour grade on glutamate levels^58^ we also compared metabolic differences between patients with, and without, seizures exclusively in those with a histological diagnosis of glioblastoma. However, Glut/Cre ratios were again not different between patients with a glioblastoma and a history of pre-operative seizures (mean = 0.18 ± 0.19, *n* = 8) and glioblastoma patients without seizures (mean = 0.16 ± 0.03, *n* = 3, *P* = 0.8411, two-tailed *t*-test, Fig. 6D). There was no difference observed in NAA/Cre ratios between the seizure (mean = 1.26 ± 0.34, *n* = 8) and non-seizure groups (mean = 1.66 ± 0.52, *n* =3, *P* = 0.1686, two-tailed *t*-test, Fig. 6E). Cho/Cre ratios were also not different between the seizure (median = 1.17, IQR 0.73–1.36, *n* = 8) and non-seizure groups (median = 0.81, IQR 0.65–0.99, *n* = 3, *P* = 0.3758, Mann–Whitney test, Fig. 6F).

We also compared the Glut/Cre ratio between IDH wild type (WT) patients and IDH mutant positive patients, where IDH mutant status was available. There was no difference between Glut/Cre ratios between IDH WT patients (median = 0.11, IQR 0.03–0.21, *n* = 10) and IDH mutant patients (median = 0.10, IQR 0.07–0.14, *n* = 13, *P* = 0.8315, Mann–Whitney test, Fig. 6G).

These findings suggest that glioma-associated epilepsy may be more dependent on localised cortical hyperexcitability rather than on measurable metabolite differences, indicating that MRS-derived glutamate levels alone are not a reliable predictor of seizure occurrence in glioma patients.

## Discussion

In this study, we have demonstrated that resected cortical peri-tumoural areas that generated IIDs *ex vivo* were associated with higher Glut/Cre ratios on pre-operative MRS, compared to peri-tumoural regions from patients where the slices were IID-negative. We consider *ex vivo* IIDs a marker of epileptogenic brain. Early work showed that IIDs were limited to samples from regions with electrographic epileptic foci but not distant regions.^38^ More recent work demonstrated IIDs from peri-tumoural cortical samples in patients with gliomas, but not control tissue,^42^ again suggesting the IIDs are a feature of the tissue and not an artefact of slicing. The finding that there were more IIDs in slices with high tumour infiltration versus low infiltration supports the notion that IIDs reflect intrinsic epileptogenic properties of peri-tumoural cortex.^42^ Our experience is that human acute cortical slice preparation is a robust tool with evidence of viability for spontaneous local field potential activity up to 20 h post resection.^39^ This is consistent with other groups that have, with modifications, maintained viable slices in holding chambers for up to 4 days post resection.^59^ The elevation in glutamate on MRS was a localised phenomenon, rather than a global elevation of glutamate in the brain of patients with spontaneous IIDs, as the difference persisted when normalised to measurements made from normal appearing white and grey matter from the same region in the contralateral hemisphere. The NAA/Cre ratio, a biomarker of neuronal density and function, was similar between all peritumoral regions indicating that the association between high Glut/Cre and IID generation did not simply reflect differences in the health of the tissue.^60^ Furthermore, the similar Cho/Cre ratio between regions that generated IIDs or were IID-negative suggests similar levels of lipid turnover and thus tumour infiltration in all peri-tumoural tissue.

### High glutamate is associated with spontaneous IIDs *ex vivo*

Elevated glutamate levels on MRS in glioma patients with seizures have been described,^25^ but to the best of our knowledge, this is the first study to demonstrate that the high Glut/Cre ratio is associated with the generation of IIDs *ex vivo*. In addition, extracellular glutamate is reported to be higher in IDH wild type patients,^26,27^ but this was not borne out in the current study, which had a smaller sample size than the aforementioned studies. Interestingly, despite the presence of IIDs *ex vivo,* we did not find any association between increased glutamate on MRS and a history of seizures. There was no difference in the Glut/Cre ratio in our patient cohort between those with or without pre-operative seizures. This lack of association between high Glut/Cre and seizure history remained when we examined only the glioblastoma cases. Our finding is consistent with previous MRS studies that also found no correlation between glutamate signals within the tumour and the incidence of pre-operative seizures.^26,27^

One explanation for these different results between glutamate levels and seizure history may reflect the nature of the metabolite signal measured on MRS in different studies. Previous studies used MRS sequences that do not distinguish glutamine from glutamate^25-28^ and have used a combined glutamate and glutamine (Gln) signal^30^ (often defined as Glx), whereas in this study, using a TE-averaged PRESS sequence, glutamate only is measured in the Glut/Cre ratio. One interpretation of the differences observed with this work and other published works^25-27^ is that it is the glutamine signal, rather than the glutamate signal, that is accounting for the differences described. Supporting this, Gottshalk *et al.*^61^ reported on patients with WHO grade 2 to 4 gliomas and compared metabolite signals in tumour volume to those in parietal white matter in healthy controls. Using a short TE PRESS sequence, the authors reported that glutamine levels were significantly higher in tumour tissue compared to healthy parietal white matter. Glioblastoma cells are known to accumulate glutamine.^62,63^ In support of this, using ^1^H high-resolution magic angle spinning nuclear magnetic spectroscopy (^1^H HRMAS NMR) Ekici *et al.*^58^ reported higher glutamine levels in tissue samples from deceased patients with gliomas compared to surviving patients in their cohorts. Of interest, low or undetectable levels of glutamine synthetase in gliomas are associated with better survival and epilepsy,^64^ which would go against increased glutamine levels in patients with seizures. To resolve these issues, further studies are needed to contrast Glut/Cre ratios and Gln/Cre ratios between patients with seizures and without seizures. Given the references to glutamine metabolism in gliomas, it may be useful to further explore the potential role of glutamine in epileptogenesis and distinguish it from glutamate effects in future studies. Lastly, in this work, Glut/Cre levels were estimated at the cortical/subcortical level rather than in white matter, which may not permit a direct comparison with the aforementioned studies, as Glut/Cre ratios are significantly higher in grey matter in normal subjects compared to white matter, and Gln/Cre ratios lower.^65^

There is some evidence for lobar differences in metabolite levels in healthy adults with a descending gradient of choline from frontal to parietal and occipital lobes, and elevated NAA in occipital lobes compared to frontal and parietal lobes.^66^ Because of the small sample size in our study, it was not possible to control for the lobe from which the samples were taken. However, to date, the authors have not found any studies that have examined regional differences in metabolites in the context of gliomas.

The absence of a difference between seizure patients and non-seizure patients may reflect the fact that elevated glutamate is found in the context of localized cortical hyperexcitability rather than being a predictor of seizure occurrence. Cortical hyperexcitability may precede the occurrence of clinically recognised seizures^67^ and may be associated with seizure-naïve patients.^68^ In a gene variant mouse model of glioblastoma, EEG recordings showed spike discharges at P45-55, with clinical seizures at P60-P80.^67^ An ECoG study of patients undergoing craniotomy for glioma resection found no differences in the rate of epileptiform discharges or high frequency oscillations (HFOs) between patients with a clinical history of seizures and seizure naïve patients.^68^ However, another study demonstrated HFOs exclusively in patients with a history of seizures,^69^ and using *ex vivo* human cortical slice recordings, IIDs have also been described in samples from patients without a history of seizures.^42^ All seizure patients were on anticonvulsants pre-operation (Supplementary Table 1). Data are limited on the effect of anticonvulsant use and peri-tumoural glutamate concentrations; one pilot study found no association between tissue concentration of Levetiracetam or Perampanel and glutamate as measured by mass spectrometry.^70^

### How does high glutamate link to the generation of spontaneous IIDs?

We found a clear link between increased cortical glutamate levels and the generation of spontaneous IIDs *ex vivo,* which could be due to an increased number of glutamate-secreting glioma cells in these peri-tumoural regions. If this were the case, we may have expected to see differences in the NAA/Cre or Cho/Cre ratios as biomarkers of neuronal health and tumour invasion, respectively. However, we found that both NAA/Cre and Cho/Cre ratios were similar between regions that generated IIDs *ex vivo,* and those that were IID-negative, suggesting cell health and tumour invasion were not markedly different between the regions. Alternatively, differences in the expression levels of different glutamate transporters could explain the high levels of glutamate, leading to spontaneous IIDs.^18,20-22,71^ These transporters include excitatory amino acid transporters (EAAT) on astrocytes and the cysteine–glutamate antiporter system x_c-_.^22^ Work done by Sontheimer’s group established that induced epileptiform-like events in glioma-implanted mouse brain slices were sensitive to the known system x_c-_ antagonist sulfasalazine.^22^ It would be interesting in future work to establish the role of system x_c-_ in human cortical glioma tissue.

### High glutamate levels on MRS as a possible biomarker to guide surgical resection

The association that we observed between elevated glutamate with the presence of spontaneous IIDs, as recorded *ex vivo* suggests that elevated glutamate on pre-operative MRS may serve as a biomarker for the mapping of epileptogenic cortex around gliomas and highlights the potential of MRS as an adjunct in guiding surgical resection to improve seizure control in patients post-operatively.

The limitation of our study was a relatively modest sample size, which did not permit detailed subgroupings, for instance, by cortical region and grade of glioma. Future work should examine this to establish the generalizability of the findings from this study.

### Future directions

The association of elevated glutamate, as measured by MRS, with spontaneous IIDs requires confirmation in patients *in vivo.* This could potentially be achieved by acquiring MRS glutamate measurements, as described in this work, pre-operatively and placing a subdural ECoG grid intra-operatively to record the presence or absence of spontaneous IIDs, with the location of the electrodes mapped to the cortical voxels defined by MRS. If the same association holds true *in vivo*, further work should clarify whether 3D MRS techniques^72^ could be used to map regions of high glutamate in the peri-tumoural regions pre- and post-operatively. Comparing the degree of postoperative seizure control with the degree of resection of regions of elevated glutamate would establish whether there is potential for a randomized controlled trial on the use of MRS-guided resection of peri-tumoural regions with elevated glutamate for seizure control.

## Supplementary Material

fcaf451_Supplementary_Data

## Data Availability

All data are available in the main text, figures and tables.
