# Supplementary material for: Elevated glioma-related cortical glutamate in vivo is associated with ex vivo interictal discharges in human brain slices
Source: Brain Commun. 2025 Nov 17;7(6):fcaf451. doi: 10.1093/braincomms/fcaf451 (PMC12674171; doi:10.1093/braincomms/fcaf451)
Supplement: fcaf451_Supplementary_Data [file fcaf451_supplementary_data.docx]

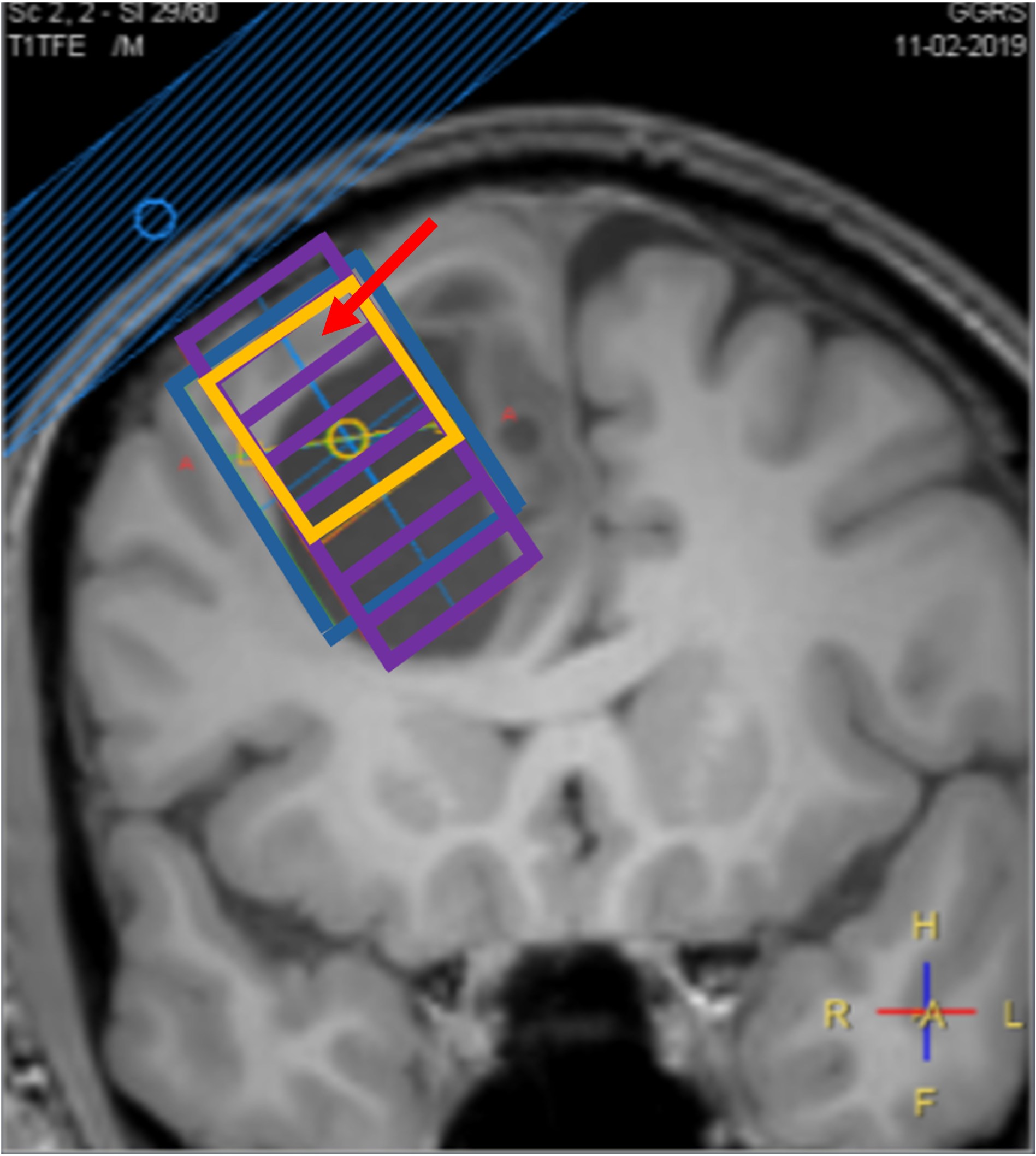


Supplementary figure 1 Example of volume of interest placement. T1 weighed coronal image of study participant with a right frontal glioma, with acquisition set up for MRS. CSI grid (purple grid) is placed with the grid orthogonal to the cortex. A VOI (orange box) from which the MRS signal is excited is then placed to include the cortex identified for biopsy (red arrow). A saturation band (shaded blue rectangle) eliminates any unwanted lipid signal from scalp fat, and active shimming (blue box) reduces any inhomogeneities in the magnetic field strengths in the volume of interest.

Supplementary table 1 Individual patient demographics

|  |  |  |  | **Patient Characteristics** |  |  |
| --- | --- | --- | --- | --- | --- | --- |
| **Study ID** | **Age** | **Sex** | **Lobe** | **Histology** | **Seizure occurrence** | **Anticonvulsant** |
| MRS + EP 1 | 54 | M | Frontal | Anaplastic astrocytoma | Yes | Levetiracetam |
| MRS + EP 2 | 66 | M | Frontal | Anaplastic oligodendroglioma | Yes | None |
| MRS + EP 3 | 60 | M | Frontal | Glioblastoma | Yes | Levetiracetam |
| MRS + EP 4 | 41 | M | Frontal | Anaplastic oligodendroglioma | No | None |
| MRS + EP 5 | 71 | M | Temporal | Glioblastoma | No | None |
| MRS + EP 6 | 48 | F | Temporal | Anaplastic oligodendroglioma | Yes | Levetiracetam |
| MRS + EP 7 | 74 | M | Frontal | Glioblastoma | Yes | Lamotrigine |
| MRS + EP 8 | 39 | M | Occipital | Glioblastoma | No | None |
| MRS + EP 9 | 67 | F | Temporal | Glioblastoma | Yes | Levetiracetam |
| MRS + EP 10 | 47 | M | Frontal | Glioblastoma | Yes | Levetiracetam |
| MRS + EP 11 | 83 | M | Temporal | Glioblastoma | Yes | Lamotrigine |
| MRS + EP 12 | 58 | M | Temporal | Anaplastic astrocytoma | Yes | Levetiracetam |
| MRS + EP 13 | 47 | M | Occipital | Glioblastoma | No | None |
| MRS + EP 14 | 49 | M | Temporal | Glioblastoma | Yes | Levetiracetam |
| MRS + EP 15 | 38 | M | Frontal | Glioblastoma | Yes | Phenytoin & Levetiracetam |
| MRS + EP 16 | 35 | M | Frontal | Oligodendroglioma | Yes | None |
| MRS + EP 17 | 63 | M | Frontal | Low grade diffuse glioma | Yes | Levetiracetam |
| MRS + EP 18 | 53 | F | Parietal | Glioblastoma | Yes | Levetiracetam |
| MRS + EP 19 | 38 | M | Frontal | Low grade diffuse glioma | Yes | None |
| MRS + EP 20 | 25 | M | Parietal | Anaplastic astrocytoma | Yes | Levetiracetam & Clobazam |
| MRS alone 1 | 40 | M | Frontal | Low grade diffuse glioma | No | None |
| MRS alone 2 | 63 | F | Frontal | Low grade diffuse glioma | Yes | Lamotrigine |
| MRS alone 3 | 27 | M | Occipital | Low grade diffuse glioma | Yes | Levetiracetam |
| MRS alone 4 | 69 | M | Insular | Radiological diagnosis LGG | Yes | Levetiracetam |
| MRS alone 5 | 49 | F | Temporal | Low grade diffuse glioma | Yes | Levetiracetam |
| Healthy volunteer 1 | 24 | F | Frontal | n/a | n/a | n/a |
| Healthy volunteer 2 | 30 | F | Occipital | n/a | n/a | n/a |
| Healthy volunteer 3 | 34 | M | Temporal | n/a | n/a | n/a |
| Healthy volunteer 4 | 24 | M | Temporal | n/a | n/a | n/a |
| Healthy volunteer 5 | 27 | M | Parietal | n/a | *n/a* | n/a |

**Full list of demographic and clinical details for each study subject. MRS + EP: study subjects with MRS and ex vivo electrophysiology data sets available. MRS alone: study subjects with MRS data sets alone.**
